# Randomised controlled trials of occupational therapy interventions for adults with a mental health condition or dementia: A systematic review of study methods and outcome measurement

**DOI:** 10.1177/03080226221086206

**Published:** 2022-04-26

**Authors:** Mary Birken, Jennifer Wenborn, Catriona Connell

**Affiliations:** 1 Division of Psychiatry, 4919 University College London, London, UK; 2 Salvation Army Centre for Addiction Services, 7622 University of Stirling, Stirling, UK

**Keywords:** review, research methods, dementia, mental health

## Abstract

**Introduction:**

High-quality randomised controlled trials (RCTs) of interventions are essential for determining whether an intervention is effective. However, many RCTs that examine the effectiveness of occupational therapy interventions for adults with mental health conditions or dementia have methodological limitations that reduce confidence in their results. We aimed to systematically review the quality of methods and outcome measures used in RCTs of occupational therapy interventions for adults with a mental health condition or dementia. This will inform future research in this area and enable practitioners to appraise the evidence when selecting interventions.

**Method:**

We searched peer-reviewed English language publications from 2000 to 2021 in MEDLINE, PsycINFO, ASSIA, CINAHL and e-thos, and hand-searched 12 journals. We included papers that met pre-specified inclusion criteria, appraised quality using a validated tool and extracted data. We conducted a narrative synthesis.

**Results:**

Of thirty-three included papers, 26 reported full or pilot RCTs, two reported secondary analysis or secondary outcomes of included RCTs, three reported process evaluations and two reported economic evaluations. Methodological limitations were found in many studies and outcome measures varied in their psychometric quality.

**Conclusion:**

High-quality RCTs of occupational therapy interventions are needed for adults with mental health conditions and dementia. Researchers should follow international guidelines for rigorously developing and evaluating interventions and reporting studies. Practitioners should critically apply RCT evidence when selecting occupational therapy interventions.

## Introduction and literature review

### Evidence-based practice

Evidence-based practice is a fundamental tenet of occupational therapy, vital for ensuring that practice is ethical, likely to produce benefit rather than harm, and delivered in a way that optimises resources (Health and Care Professions Council, 2013). This requires all occupational therapists to engage with research (Royal College of Occupational Therapists [RCOT], 2019). When selecting an intervention, occupational therapists must apply the best available evidence that an intervention is effective for the outcome the service user seeks to achieve. They must then integrate this with their knowledge of the context and person with whom they are working (RCOT, 2021).

### Randomised controlled trials and why they matter

The strongest, or ‘gold standard’, evidence that an intervention is effective is when it has been tested and proven to be so in a fully powered randomised controlled trial (RCT), that is, the trial recruits a large enough sample to detect a difference on primary outcome measure, between the two groups if such a difference exists. Effectiveness has been defined as: How beneficial treatment is under usual conditions, compared with doing nothing or opting for another type of treatment (National Institute for Health and Care Excellence [NICE], 2021). Evidence of effectiveness is further strengthened when studies’ data are combined in a meta-analysis. However, this requires comparability between studies in terms of the population, intervention and outcomes being examined. RCT evidence is critical for service level decisions.

The UK National Institute for Health and Care Excellence (NICE) produces evidence-based guidance for health and social care. Interventions are recommended if there is RCT evidence of both clinical and cost effectiveness. NICE guidelines recommend occupational therapy intervention in some practice areas, including people living with dementia and their carers (NICE, 2018). However, occupational therapy is not included in any mental health guidelines due to a lack of robust RCT evidence. The implication of this omission is that occupational therapy is not deemed a funding priority by service commissioners, thus denying service users access to it.

### Guidance for conducting and reporting RCTs

We can only evaluate evidence that is published. Further, if a high-quality study is poorly reported, its findings may not be appreciated. Reporting guidelines support the transparent and accurate conduct and reporting of research activities, as well as guiding study design. The international Enhancing the QUAlity and Transparency Of health Research Network (n.d.) (EQUATOR Network) has published a series of evidence-based recommendations for reporting research, including the Consolidated Standards of Reporting Trials (CONSORT) Statement for RCTs (Schulz et al., 2010).

Conducting high-quality research has cost and resource implications but is essential to avoid the resource waste, and potential harms, in practice of delivering ineffective interventions selected based on poor quality evidence. In the United Kingdom, health research funders expect applicants to use the Medical Research Council (MRC) and NIHR framework for developing and evaluating complex interventions (Skivington et al., 2021). This describes the integrated phases: identifying/developing an intervention; testing the feasibility of the intervention and evaluation design, including the outcome measures; running a rigorous full evaluation and implementation study if proven successful. The new guidance includes economic and contextual considerations at all phases, commonly seen in economic and process evaluations. This approach reduces costly research waste caused by inadequately developed interventions and unclear intervention outcomes being tested in RCTs.

### Economic and process evaluations

Economic evaluation alongside RCTs is essential to inform decisions to implement an intervention in practice. Economic evaluation compares the costs and outcomes (or consequences) between two or more interventions and verifies the cost consequences of choosing one intervention with evidence of effectiveness over another (Razzouk, 2017).

Process evaluation seeks to understand how the intervention works (mechanisms of impact), in which context it works best, and how it is delivered in the research (implementation). Results contribute to interpreting RCT results and their generalisability (Moore et al., 2014), and determining how to put interventions into practice. Process evaluation uses quantitative and qualitative methods depending on the questions at each stage. For example, understanding the intervention mechanisms of impact through qualitative interview data is important at the feasibility and pilot stages to refine the intervention content and delivery prior to a full RCT. Assessing the fidelity of delivery by analysing quantitative data, for example, number of sessions delivered as intended, is important in a full RCT.

### Quality of RCTs of occupational therapy interventions in mental health and dementia

The main foci of occupational therapy are ‘to enable people to participate in the activities of everyday life’ and to achieve optimal ‘health and wellbeing through occupation’ (WFOT, 2010). Occupational therapy interventions for people with mental health conditions or dementia reflect this occupation-centred approach. Occupation-centred interventions are defined as: interventions where information about the person, environment and occupation relates closely with occupational performance and where the ‘doing’ of occupation is the main ingredient in the intervention and in the outcomes measured (Fisher, 2013). However, for people with mental health conditions or dementia there are few RCTs of occupation-centred interventions with primary outcome measures that consider participation in everyday activities. Kirsh et al. (2019) identified that further research involving rigorous study designs are needed to advance the evidence base for occupational therapy interventions for adults with mental health conditions, as current published studies are underpowered and pilot studies often do not progress to fully powered RCTs. A systematic review and meta-analysis of the effectiveness of occupational therapy provided at home to people with dementia and their family caregivers demonstrated that the evidence was of very low to moderate quality (Bennett et al., 2019). There was a high risk of performance bias caused by small sample sizes, inconsistent results between studies and lack of blinding of participants, therapists and assessors (Bennett et al., 2019).

Tokolahi and colleagues (2015) reviewed the characteristics, quality and reporting of 14 cluster RCTs of occupational therapy interventions. Cluster RCTs are trials in which groups of individuals rather than individuals themselves are randomly allocated to different intervention arms (Eldridge and Kerry, 2012). The review identified that involving a statistician was associated with improved trial quality and reporting. They recommended more detailed reporting of cluster RCTs to facilitate accurate appraisal of the quality of the findings, and increased reporting of intraclass correlation coefficients to improve credibility of results (Tokolahi et al., 2015). However, as this review did not include other types of RCT design, a systematic review investigating the methods and outcomes used in randomised controlled trials of occupational therapy interventions for people with diagnosed mental health conditions or dementia is needed.

## Aim

To improve future RCTs of occupational therapy interventions for adults with mental health conditions or dementia, a crucial first step is to systematically identify the methodological strengths and weaknesses common in the literature. This is distinct from reviewing evidence of effectiveness, which has already been established as limited.

We systematically review and evaluate the quality of the methods and outcome measures used in RCTs of occupational therapy interventions for adults with mental health conditions or dementia to:

- Identify common methodological practices, strengths and weaknesses. This will maximise the likelihood that future research is of sufficient quality and comparability to draw definitive conclusions.
- Synthesise characteristics of studies and appraise their quality. This will support practitioners when selecting and applying evidence to support their practice.

### Review question

What are the optimal methods and outcomes to use when conducting randomised controlled trials of occupational therapy interventions for adults with a diagnosed mental health condition or dementia?

## Method

### Design

We registered this systematic review in advance with PROSPERO (CRD42020183567) available from: https://www.crd.york.ac.uk/prospero/display_record.php?ID=CRD42020183567.

**Figure 1.**
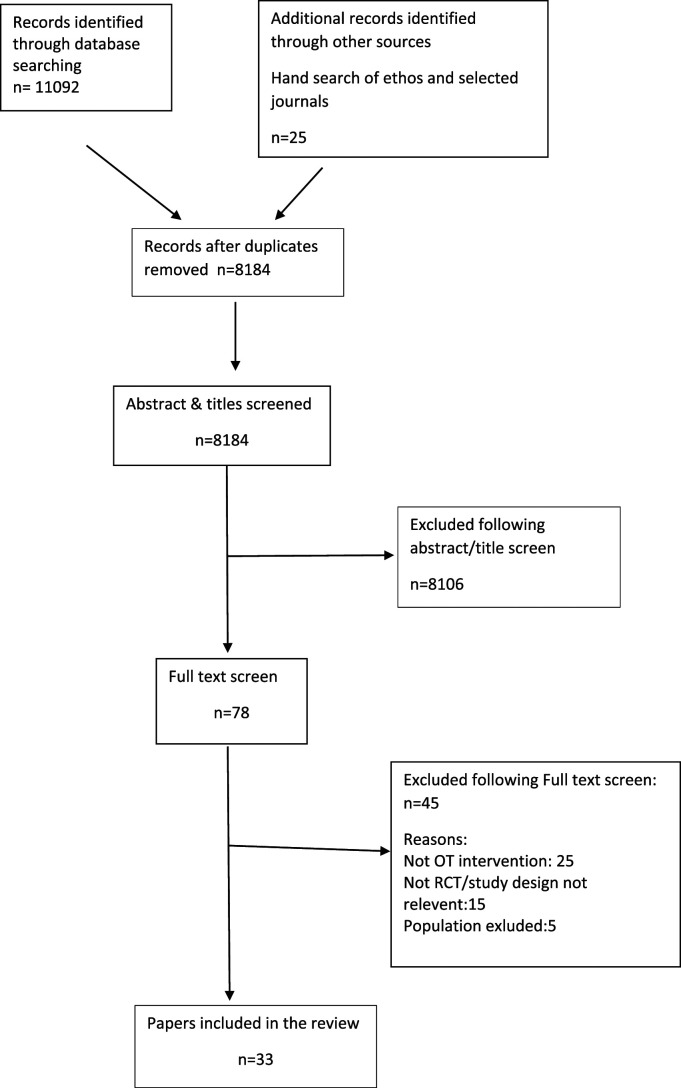
PRISMA flow diagram of papers through the review.

We include a PRISMA flow diagram to report our search results (Moher et al., 2009) (see Figure 1).

### Eligibility

We included studies where the population is over 18 years old and diagnosed with a mental health condition or dementia. We excluded studies where the population lived in nursing home or continuing care settings, as the authors considered that the methodological challenges in conducting intervention research in this setting were beyond the scope of this review. Studies were also excluded if the intervention was provided to carers only (formal or informal) as we were interested in the methodological issues arising from involving adults with mental health conditions or dementia in occupational therapy RCTs.

We included occupation-centred interventions, as defined above, delivered by an occupational therapist or a worker supervised by an occupational therapist, and provided within the context of an occupational therapy service. We included interventions delivered to the individual with a diagnosed mental health condition or dementia, and those that included an informal carer such as a family member within a dyadic intervention. We excluded psychological (e.g. Cognitive Stimulation Therapy and Cognitive Behavioural Therapy) and pharmacological interventions as they are not occupation-centred.

We included all comparators and all intended outcomes of interventions as stated in paper, reflecting our aim to appraise the methods, outcomes and their measurement, rather than intervention effectiveness.

We included feasibility RCTs, pilot RCTs, full RCTs and economic evaluations and process evaluations conducted within or alongside RCTs.

### Search strategy

We combined terms for occupational therapy, with terms for mental health or dementia, and terms for RCT, fidelity, process evaluation, implementation and cost effectiveness or economic evaluation. We searched agreed terms mapped and unmapped against subject headings (see Appendix 1, for example).

We conducted electronic searches in MEDLINE, PsycINFO, ASSIA, CINAHL and e-thos. We hand-searched twelve key journals in the fields of: implementation science, occupational therapy, mental health and dementia.

We screened the reference lists of the identified studies for other eligible studies.

We limited our searches to studies published from 2000 onwards, because occupational therapy RCTs prior to 2000 are likely to be few, of low quality and with high risk of bias due to a lack of international standards at that time. We also limited our searches to studies reported in English.

### Study selection

We imported the search results into EndNote and removed duplicates. One author (MB) screened all the abstracts and titles and removed references considered to be irrelevant. A second author (CC) screened 20% of the titles and abstracts to check reliability. (MB) and (CC) independently screened the full text of the remaining studies before comparing results. Where there were disagreements, the third author (JW) screened the full text and made the final decision.

### Data extraction

We divided the studies between us and extracted the data using a predefined data extraction form based on relevant CONSORT headings and all Downs and Black checklist items (Downs and Black, 1998). Items included, but were not limited to, the recruitment and randomisation processes used, masking of participants, the interventions for each group and sample size.

### Methodological Quality Assessment

The Downs and Black checklist (1998) comprises 27 items, each with a numeric rating up to a maximum score of 28. It assesses the quality of reporting, external validity, internal validity, and power. We assigned a quality descriptor to each study based on the raw score as follows: ‘poor’ (<14 points), ‘fair’ (14–18), ‘good’ (19–23) and ‘excellent’ (24–28) (O’Connor et al., 2015).

### Data synthesis

We determined strengths and limitations at study level and appraised methodological quality across all studies by tabulating and comparing items of the Downs and Black checklist and overall rating. We tabulated the outcome measures used and defined the outcome domain(s) assessed. We reviewed the occupation-focused measures, grouped together those measuring the same outcome and noted the number of studies adopting each measure. We appraised the stated primary outcomes for theoretical coherence to the intervention(s) tested (i.e. whether they measured what the intervention intended to change). We determined whether authors had cited studies assessing the outcome measures’ psychometric properties. We noted if authors reported sample size calculation based on the study’s primary outcome measure. We planned to use thematic analysis to synthesise the reported barriers and facilitators to conducting RCTs in this field. However, the lack of process evaluation data made this unviable. Instead, we conducted a narrative synthesis to summarise the quality ratings, methods, intended aims of interventions and outcome measures used within the studies.

## Results/findings

Following removal of duplicates, we identified 8184 papers from the searches. We screened the full text of 78 papers, resulting in inclusion of 33 papers reporting 26 studies (see Figure 1).

Table 1 summarises the included papers’ characteristics. Articles were published between 2001 and 2021. Studies took place in thirteen countries, with the most conducted in Brazil (*n* = 4), USA (*n* = 4), Taiwan (*n* = 3) and the Netherlands (*n* = 3). Nine studies were reported as being pilot RCTs: six were conducted with adults with mental health conditions and three with people with dementia. Seventeen studies were reported as being RCTs: ten were conducted with adults with mental health conditions and seven with people with dementia. Two papers reported secondary analysis or secondary outcomes of RCTs included in this review. Three papers reported process evaluations and two reported economic evaluations conducted within the pilot or full RCTs.

**Table 1.** Summary of papers included in review (*n* = 33).

Study			Methods		Population				Interventions				Quality
Author, publication year	Country	Study design	Sample size calculation (primary outcome)	Sample size	Participants’ diagnosis/condition	Age (mean years)	Ethnicity (%)	Gender (female %)	Name (acronym)	Intervention: level of description	Intervention fidelity assessed	Comparator	Grade
Abaoğlu et al. (2020)	Turkey	RCT	NR	38	Schizophrenia	39.57	NR	40.63	Individualised Life Skills training	C	NR	C	Poor
Argentzell et al. (2020)	Sweden	Mixed model regression analysis of Cluster RCT data, results of which previously reported by Eklund et al., 2017 (see below)											
Buchain et al. (2003)	Brazil	RCT	NR	26	Treatment-resistant schizophrenia	35	NR	26.9%	Occupational Therapy	A	NR	A	Fair
Callahan et al. (2017)	USA	RCT	Y	180 [pairs]	Alzheimer’s disease	78.41	56.66 Black	70.55	Home-based Occupational Therapy [dyadic]	B, E	NR	A	Fair
Chen et al. (2015a)	Taiwan	RCT	N	68	Major depressive disorder	48.85	NR	73.5	Life Adaptation Skills Training (LAST)	B, C	NR	C	Fair
Chen et al. (2015b)	Taiwan	Pilot RCT	N	21	Mood disorder	44.9	NR	66.7	Quality of Life Enhancement Programme (QOLEP)	B, C	NR	C	Fair
Cook et al. (2009)	UK	Pilot RCT	N	44	Schizophrenia/Psychosis	39	White British: 77	34.09	Community-based Occupational Therapy	B, E	OT adherence monitored	A	Good
Edgelow and Krupa (2011)	Canada	Pilot RCT	N	24	Serious mental illness	39.17	NR	NR	Action over Inertia (AOI)	B, E	NR	A	Fair
Eklund et al. (2017)	Sweden	Cluster RCT	Y	226	Mental illness	40	NR	72	Balancing Everyday Life (BEL)	C, D	Self-rated by OT	A	Fair
Foruzandeh and Parvin (2013)	Iran	Pilot RCT	N	60	Chronic schizophrenia	38.67	NR	28.13	Occupational Therapy	C	NR	A	Poor
Gitlin et al. (2008)	USA	Pilot RCT with wait-list control	N	60 [pairs]	Dementia	79.4	White: 76.7	43.3	Tailored Activity Program (TAP) [dyadic]	C, E	OT recorded dosage and adherence	A	Good
Gitlin et al. (2009)	USA	Process evaluation conducted alongside pilot RCT reported as Gitlin et al., 2008 (see above)											
Gitlin et al. (2010)	USA	Economic evaluation conducted alongside pilot RCT reported as Gitlin et al., 2008 (see above)											
Gitlin et al. (2018)	USA	RCT	Y	160 [pairs]	Dementia	80.4	White: 79.4	3.1	Tailored Activity Program – Veterans version (TAP-VA) [dyadic]	B, E	Adherence checklists	C	Good
Graff et al. (2006)	The Netherlands	RCT	Y	135 [pairs]	Mild to moderate dementia	78.11	NR	55.5	Community Occupational Therapy in Dementia (COTiD) [dyadic]	B, D, E	NR	A	Good
Graff et al. (2007)	The Netherlands	RCT: reports secondary outcomes of RCT conducted by Graff et al., 2006 (see above)											
Graff et al. (2008)	The Netherlands	Economic evaluation conducted alongside pilot RCT reported as Graff et al., 2006 (see above)											
Grimm et al. (2009)	USA	Pilot RCT	N	8	Schizophrenia	43.8	62% White 25% African American 13% Korean	25	Occupational Therapy using acquisitional frame of reference	C	NR	A	Poor
Gunnarsson et al. (2018)	Sweden	RCT	Y	121	Depression and/or anxiety	41.6	NR	83	Tree Theme Method	B	NR	B	Fair
Hees et al. (2013)	The Netherlands	RCT	NR	117	Major depressive disorder	43	NR	51	Occupational Therapy	C, E	NR	A	Good
Kim (2020)	Korea	RCT	NR	35	Mild Alzheimer’s disease	79.2	NR	74	Recollection-Based Occupational Therapy Program	C, D	NR	A	Poor
Kumar et al. (2014)	India	RCT	NR	77	Mild to moderate dementia	69.39	NR	19.5	Occupational Therapy Program (OTP)	C, E	NR	A	Fair
Novelli et al. (2018)	Brazil	Pilot RCT with wait-list control	Y	30 [pairs]	Dementia	81.37	NR	50	Tailored Activity Program – Brazilian version (TAP-BR) [dyadic]	B, E	OTs trained and supervised	NR	Fair
Oliveira et al. (2018)	Brazil	Pilot RCT	N	21 [pairs]	Dementia	78.7	NR	71.4	Tailored Activity Program - Outpatient Version (TAP-O) [dyadic]	C, E	NR	C	Fair
Schene et al. (2007)	the Netherlands	RCT & economic evaluation	NR	62	Major depressive disorder	45.87	NR	51.6	Occupational Therapy	B, D	NR	A	Fair
Shimada et al. (2018)	Japan	RCT	Y	136	Schizophrenia or schizoaffective disorder	42.68	NR	50.73	Individualised occupational therapy (IOT)	B, C	NR	A	Poor
Tatsumi et al. (2012)	Japan	RCT	NR	38	Schizophrenia	57.6	NR	47	Occupational therapy	B	NR	A	Fair
Vizzotto et al. (2016)	Brazil	Pilot RCT	N	25	Treatment-resistant schizophrenia	38.6	NR	17	Occupational Goal Intervention (OGI)	A	NR	C	Fair
Voigt-Rafloff et al. (2011a)	Germany	Process evaluation conducted alongside RCT reported as Voigt-Radloff et al., 2011b (see below)											
Voight-Radloff et al. (2011b)	Germany	RCT	Y	141 [pairs]	Mild to moderate dementia	(77.8)	NR	58	Community Occupational Therapy in Dementia – German translation (COTiD) [dyadic]	C, D, E	Y – see Voigt-Radloff et al., 2011a	C	Excellent
Walton et al. (2019)	UK	Process evaluation (fidelity) conducted alongside RCT reported as Wenborn et al., 2021 (see below)											
Wenborn et al. (2021)	UK	RCT	Y	468 [pairs]	Mild to moderate dementia	78.6	White British: 91	43	Community Occupational Therapy in Dementia - UK version (COTiD-UK) [dyadic]	C	Y – see Walton et al., 2019	A	Excellent
Wu (2001)	Taiwan	RCT	NR	166	Mental illness	36.8	NR	49	Occupational therapy using motivational frame of reference	A	NR	B	Poor

N = No; NR = Not reported; OT = Occupational Therapist; Y = Yes; [dyadic], an intervention that includes both the person with dementia and a family carer/supporter taking part together.

Intervention: level of description, A = Very Brief description: ≤ four sentences, does not describe format, number of sessions. B = Brief description: paragraph, describes format, number of sessions; C = Session content described: text or Table; D = Intervention manual noted; E = Reference to fuller description provided

Comparator, A = ‘Treatment as usual’ may or may not include occupational therapy; B = ‘Standard’ occupational therapy, as usually provided; C = ‘Other’ intervention, for example: activity sessions, telephone contacts.

Four interventions for people with dementia were reported across more than one paper. Three papers referring to the Tailored Activities Program (TAP) report pilot trial, the process evaluation and economic evaluation (Gitlin et al., 2008, 2009, 2010). Three papers referring to the Community Occupational Therapy in Dementia (COTiD) in the Netherlands, report primary and secondary outcomes and the economic evaluation (Graff et al., 2006, 2007, 2008). Two papers report the process evaluation and outcomes of the German trial of COTiD (Voigt-Rafloff et al., 2011a; 2011b). Two papers report the outcomes (Wenborn et al., 2021) and fidelity of delivery (Walton et al., 2019) of the UK version of COTiD (COTiD-UK).

Of the 28 papers reporting pilot or full RCTs, and secondary analysis or secondary outcomes of RCTs, five did not include the study design within the title or abstract. All titles or abstracts stated the study population but two did not name the intervention. Only six titles or abstracts included the term ‘occupational therapy’.

The pilot trials recruited between eight and 60 individuals or between 21 and 60 pairs for the dyadic interventions. The 17 RCTs, recruited 26–226 individuals and 135–468 pairs of people with dementia and their family carers. Eight reported a power calculation to determine the sample size required to detect change between the groups (Callahan et al., 2017; Eklund et al., 2017; Gitlin et al., 2018; Graff et al., 2006; Gunnarsson et al., 2018; Shimada et al., 2018; Voight-Radloff et al., 2011b; Wenborn et al., 2021). Eklund et al. (2017) performed their power calculation with the Satisfaction with Daily Occupations (SDO). However, in their study implementation, Eklund et al. (2017) combined the SDO with another question (one item of the occupational balance questionnaire) which was not part of the power calculation.

### Quality assessment

We extracted data from all 33 papers in rating the quality of the 26 trials. Of the pilot RCTs, two were rated as poor, five were fair and two were good. Of the RCTs, four were rated poor, eight fair, three good, and two excellent.

Internal validity is the extent to which the study is free from bias. Performance bias refers to participants being ‘masked’ or ‘blinded’ to the intervention they have received. All except one (Eklund et al., 2017) of the pilots and RCTs scored ‘0’ on this quality assessment item, meaning it was either reported that participants were not masked to their allocation or we were unable to determine this. There were examples of those delivering the intervention or comparator not being informed of its aim, or not being told which was the intervention and comparator, or the difference between the groups. Detection bias is mitigated by those collecting outcome data being masked to group allocation. Six pilot RCT studies (Cook et al., 2009; Edgelow and Krupa, 2011; Gitlin et al., 2008; Novelli et al., 2018; Oliveira et al., 2018; Vizzotto et al., 2016) and eleven RCTs reported this (Callahan et al., 2017; Chen et al., 2015a; Eklund et al., 2017; Gitlin et al., 2018; Graff et al., 2006; Gunnarsson et al., 2018; Hees et al., 2013; Schene et al., 2007; Voight-Radloff et al., 2011b; Wenborn et al., 2021; Wu, 2001). We were unable to determine this in the remaining studies. The two studies rated excellent (Voight-Radloff et al., 2011b; Wenborn et al., 2021) were both reported across two papers, that is process evaluations and RCTs, indicating good reporting of findings.

### Study participants

Demographic information was largely restricted to age and gender; only six studies reported participants’ ethnicity (Callahan et al., 2017; Cook et al., 2009; Gitlin et al., 2008, 2018; Grimm et al., 2009; Wenborn et al., 2021). Eight of the pilot or RCTs recruited pairs or dyads consisting a person with dementia along with a family carer/supporter who took part in the study and the intervention together. Demographic data is required for determining external validity, that is, the degree to which the sample is representative of the population sampled and from which inferences are made. This enables readers to appraise if the results are relevant to the people with whom they work.

### Interventions

Description of the interventions varied. Twelve were called occupational therapy but none describe the same content (Buchain et al., 2003; Callahan et al., 2017; Cook et al., 2009; Foruzandeh and Parvin, 2013; Grimm et al., 2009; Hees et al., 2013; Kim, 2020; Kumar et al., 2014; Schene et al., 2007; Shimada et al., 2018; Tatsumi et al., 2012; Wu, 2001). Another seven interventions were reported (Abaoğlu et al., 2020; Chen et al., 2015a, 2015b; Edgelow and Krupa, 2011; Eklund et al., 2017; Gunnarsson et al., 2018; Vizzotto et al., 2016). Four versions of TAP were reported (Gitlin et al., 2008, 2018; Novelli et al., 2018; Oliveira et al., 2018) and three versions of (COTiD) (Graff et al., 2006; Voigt-Radloff et al., 2011b; Wenborn et al., 2021). We summarised the level of description provided using the following categories, with some papers included in more than one category: (1) Very brief description consisting of up to four sentences, does not describe the format, nor number of sessions; (2) Brief description consisting at least one paragraph, describing the format and number of sessions; (3) Session content described in the text and/or a Figure/Table; (4) Intervention manual reported and (5) Reference to a fuller description provided (see Table 1).

### Comparators

We summarised and categorised the comparators used as follows: A. ‘Treatment as usual’ or ‘usual care’ which comprised the service provided within the locality or unit where participants were recruited from. This was often multi-professional and did or did not include occupational therapy, although in some cases this level of detail was not reported; B. ‘Standard’ occupational therapy as usually provided within the locality or unit and C. ‘Other’ intervention. We categorised most studies as being A (*n* = 17), two as B, although we acknowledge this is only as accurate as the descriptions provided. Two did not report the comparator. These were both wait-list designs so the control group received the intervention once data collection was completed. The remaining seven studies used a different comparator, mainly an alternative activity-based or educational intervention without an occupational focus, using the same number of contacts or sessions (Chen et al., 2015a, 2015b; Gitlin et al., 2018; Oliveira et al., 2018; Vizzotto et al., 2016); or a single advisory session (Abaoğlu et al., 2020, Voight-Radloff et al., 2011b) (see Table 1).

### Outcomes and outcome measures

Table 2 summarises the intended outcome of intervention as stated in the paper and the primary outcome measure(s) used.

**Table 2.** Intervention aims and intended outcomes compared with primary outcome measures as reported by study.

Study	Intervention name (acronym)	Intervention aim	Intended outcome of intervention as stated in paper	Primary outcome measure	Psychometric properties for use with study population reported or cited		
					Reliability	Validity	Sensitivity to change
Abaoğlu et al. (2020)	Individualised life skills training	Gain the skills necessary to live independently and to support meaningful, productive roles	Improvement in negative symptoms of schizophrenia	The Positive and Negative Syndrome Scale (PANSS)^a^	Y	Y	Y
				The Clinical Global Impression (CGI) Scale: one domain	N	N	N
			Improvement in disease severity	The Katz Index of Independence in Activities of Daily Living (ADL)	N	Y	N
				The Lawton and Brody Instrumental Activities of Daily Living (IADL) Scale	N	N	N
			Activities of daily living	The Social Functioning Scale (SFS)	Y	Y	N
				The Functional Assessment Short Test (FAST)	Y	Y	N
Buchain et al. (2003)	Occupational Therapy	NR	NR	Scale of Interactive Observation in Occupational Therapy (EOITO)	Y	Y	N
Callahan et al. (2017)	Home-based Occupational Therapy	Support self-care functional capability [dyadic]	Delay in functional Decline	Alzheimer’s Disease Cooperative Study Group Activities of Daily Living Inventory (ADCS ADL)	Y	Y	Y
Chen et al. (2015a)	Life Adaptation Skills Training (LAST)	Enhance lifestyle rearrangement and coping skills	Quality of life	The World Health Organization Quality of Life – BREF-Taiwan version (WHOQOL-BREF-TW)^a^	Y	Y	Y
			Occupational and environmental improvement	Occupational Self-Assessment (OSA)	Y	Y	N
			Symptom reduction	Mastery scale – Chinese version	Y	Y	N
				Social Support Questionnaire-Short Form (SSQSF)	Y	Y	N
Chen et al. (2015b)	Quality of Life Enhancement Programme (QOLEP)	Improve participation in meaningful occupation to have positive influence on health and quality of life	Improvements in quality of life	World Health Organization Quality of Life – BREF-Taiwan version (WHOQOL-BREF-TW)^a^	Y	Y	Y
			Sense of competence and environmental resources				
			Sense of Mastery				
			Perception of social support available	Occupational Self-Assessment Scale (OSA) – Chinese version	Y	Y	N
			Depression Anxiety	Mastery scale – Chinese version	Y	Y	N
				Social Support Questionnaire-Short Form	Y	Y	N
			Suicidal ideation	Beck Depression Inventory-II – Chinese version	Y	Y	Y
				Beck Scale for Suicide Ideation – Chinese version	Y	Y	N
Cook et al. (2009)	Community-based Occupational Therapy	NR	Social Functioning	Social Functioning Scale (SFS)	Y	Y	Y
Edgelow and Krupa (2011)	Action over Inertia (AOI)	Improve occupational balance and engagement	Improved occupational balance and engagement	24-hr time diaries^a^	N	N	N
				Profiles of Occupational Engagement for People with Schizophrenia	Y	Y	N
Eklund et al. (2017)	Balancing Everyday Life (BEL)	Improve activity balance, activity engagement and valued and satisfying activities	Activity engagement	Profiles of Occupational Engagement among people with Severe mental Illness (POES) – Swedish version^b^	N	N	N
			Activity satisfaction and activity balance				
			Activity value	Satisfaction with daily occupations and occupational balance (SDO-OB)	N	Y	N
				Occupational Value with predefined items (Oval-pd), Swedish version	Y	N	N
Foruzandeh and Parvin (2013)	Occupational Therapy	NR	Improvement in Positive and negative symptoms of schizophrenia	Andreasen’s Scale for assessment of positive symptoms (SAPS)^a^	Y	N	N
				Andreasen’s scale for assessment of negative symptoms (SANS)	Y	N	N
Gitlin et al. (2008)	Tailored Activity Program (TAP)	Reduce behavioural and psychological symptoms of dementia [dyadic]	Reduced neuropsychiatric behaviours and caregiver burden.	Occurrence of 24 behaviours measured using combination of	N	N	N
				Agitated Behavior in Dementia Scale (ABID)			
				Revised Memory and Behavior Problem Checklist (RMBPC) + researcher/family carer experience			

Gitlin et al. (2018)	Tailored Activities Program – for Veterans (TAP-VA)	Reduce behavioural and psychological symptoms of dementia [dyadic]	Reduce behavioural symptoms and functional dependence	Neuropsychiatric Inventory—Clinician (NPI-C)	Y	Y	N
Graff et al. (2006)	Community Occupational Therapy in Dementia (COTiD)	Train a) person with dementia to compensate for cognitive decline; b) family carers in coping behaviours and supervision [dyadic]	Improvement in Daily Functioning	Assessment of Motor and Process Skills (AMPS): process scale	Y	Y	Y
Grimm et al. (2009)	Occupational Therapy using acquisitional frame of reference	Improve meal preparation	Improved occupational performance of meal preparation	Performance Assessment of Self-Care Skills (PASS-clinic)	Y	Y	N
Gunnarsson et al. (2018)	Tree Theme Method	Increase the ability to cope with, and to enhance satisfaction with everyday life	Performance of activities and satisfaction with the performance of activities in everyday life	Canadian Occupational Performance Measurement (COPM)^b^	Y	Y	Y
				Satisfaction with Daily Occupations (SDO)	Y	Y	N
			Balance between activities in everyday life	Occupational Balance Questionnaire (OBQ)	Y	Y	N
				Symptom Checklist-90-R (SCL-90-R)	Y	Y	N
			Psychological symptoms.	Hospital Anxiety and Depression Scale (HADS)	Y	Y	N
Hees et al. (2013)	Occupational Therapy	Improve occupational functioning related to work	Work participation, defined in terms of absenteeism and time until partial/full RTW	Work participation	NA	NA	NA
Kim (2020)	Recollection-Based Occupational Therapy program	NR	Cognitive Function	Functional Independent Measure (FIM)^a^	N	N	N
				Korean Mini-Mental Status Examination (K-MMSE)	N	N	N
				Subjective Memory Complaints Questionnaire (SMCQ)	N	N	N
				Short-Form Geriatric Depression Scale-K (SGDS-K)	N	N	N
				Geriatric Quality of Life-Dementia (GQOL-D)	N	N	N
Kumar et al. (2014)	Occupational Therapy program (OTP)	Maintain an optimal level of performance in daily life	Improved Quality of Life	World Health Organization Quality of Life measure (WHOQOL-BREF) – Hindi version	N	N	N
Novelli et al. (2018)	Tailored Activity Program – Brazilian version (TAP-BR)	Reduce behavioural and psychological symptoms of dementia [dyadic]	Reduced behavioural psychological symptoms of dementia (BPSD)	Neuropsychiatric Inventory (NPI)	Y	Y	Y
Oliveira et al. (2018)	Tailored Activity Program – Outpatient Version (TAP-O)	Reduce behavioural and psychological symptoms of dementia [dyadic]	Reduced neuropsychiatric symptoms (NPS)	Neuropsychiatric Inventory – Clinician rating scale (NPI-C) – Brazilian version	Y	Y	Y
Schene et al. (2007)	Occupational Therapy	Restore and improve occupational functioning relating to work	Depression	Structured Interview for the Diagnosis of DSM-IV Mood Disorders^a^	N	N	N
			Work Resumption				
				Becks Depression Inventory (BDI)	N	N	N
			Work Stress	Work Resumption Questionnaire	N	N	N
				Questionnaire Organization Stress (QOS)	N	N	N
Shimada et al. (2018)	Individualised occupational therapy (IOT)	Enhance cognitive functioning and prompt adaptive behaviours to maximise functional outcomes	Improve Cognitive functioning	Brief Assessment of Cognition in Schizophrenia Japanese version (BACS-J)^a^	N	N	N
			Improve social functioning	Schizophrenia Cognition Rating Scale Japanese version (SCoRS-J)	N	N	N
Tatsumi et al. (2012)	Occupational therapy	NR	Improve interpersonal relationships	Brief Psychiatric Rating Scale (BPRS)^a^	N	N	N
				Scale for Assessment of Negative Symptoms (SANS)	N	N	N
				Rehabilitation Evaluation Hall and Baker (Rehab) scale	N	N	N
			Reduce negative symptoms of schizophrenia	Profile of Mood States (POMS) scale	N	N	N
				Seating preference	N/A	N/A	N/A
Vizzotto et al. (2016)	Occupational Goal Intervention (OGI)	Improve Executive Functioning to improve everyday functioning	Improve Executive Functioning	Behavioural Assessment of the Dysexecutive Syndrome (BADS)	N	N	N
Voight-Radloff et al. (2011b)	Community Occupational Therapy in Dementia – German translation (COTiD)	Improve daily functioning of the person with dementia and the primary (family) carer [dyadic]	Improve daily functioning	Interview for Deterioration in Daily Living Activities in Dementia (IDDD): performance scale^a^	Y	Y	Y
				Perceive, Recall, Plan and Perform System of Task Analysis (PRPP)	Y	Y	Y
Wenborn et al. (2021)	Community Occupational Therapy in Dementia – UK version (COTiD-UK)	Improve a) person with dementia’s ability to engage in meaningful occupation; b) family carer’s sense of competence [dyadic]	Personal care and Instrumental activities of daily living performance	Bristol Activities of Daily Living Scale (BADLS)	Y	Y	Y
Wu (2001)	Occupational therapy using motivational frame of reference	Facilitate intrinsic motivation	Improvement in Intrinsic Motivation	Chinese General Causality Orientations Scale (GCOS)^a^	Y	Y	N
				Chinese Comprehensive Occupational Therapy Evaluation Scale (COTE)	Y	Y	N

N = No; N/A = Not Applicable; Y = Yes; BPSD = Behavioural and Psychological Symptoms of Dementia.

^a^ Primary outcome measure is not specified.

^b^ Multiple primary outcome measures listed.

Half (*n* = 13) the studies either named more than one primary outcome measure or listed a number of outcome measures without specifying which was the primary one.

Seventeen studies used a primary outcome measure, or at least one of several primary outcome measures, covering domains relevant to occupational therapy and occupation-centred interventions, ‘occupational outcome measures’. Twelve were mental health studies (Abaoğlu et al., 2020; Buchain et al., 2003; Chen et al., 2015a, 2015b; Cook et al., 2009; Edgelow and Krupa, 2011; Eklund et al., 2017; Grimm et al., 2009; Gunnarsson et al., 2018; Hees et al., 2013; Schene et al., 2007; Tatsumi et al., 2012). Five were studies of people with dementia (Callahan et al., 2017; Graff et al., 2006; Kim 2020; Voight-Radloff et al., 2011b; Wenborn et al., 2021). The four studies evaluating versions of TAP (Gitlin et al., 2008, 2018; Novelli et al., 2018; Oliveira et al., 2018) measured Behavioural and Psychological Symptoms of Dementia (BPSD), reflecting TAP’s key aim of reducing BPSD. The remaining studies used measures of performance components (e.g. cognitive skills, motivation), symptoms (e.g. depression) or other domains (e.g. quality of life).

We identified 26 occupational outcome measures. We grouped these according to the domains measured (see Table 3).

**Table 3.** Frequency of occupational outcome measures used.

Occupational balance (time use, range *and* meaning/satisfaction)	Occupational competence (activity performance *and* satisfaction)	Occupational value	Occupational environment (environment *and* satisfaction)	Activity performance only
Occupational Balance	Canadian Occupational Performance Measure (COPM): 1	Canadian Occupational Performance Measure (COPM) (participant rates): 1	Occupational Self-Assessment (OSA): 2	Alzheimer’s Disease Cooperative Study Group Activities of Daily Living Inventory (ACDS-ADL): 1
Questionnaire (OBQ): 1	Functional Assessment Short Test (FAST): 1	Occupational Self-Assessment (OSA) (SU rates): 2		Assessment of Motor and Process Skills (AMPS): 1
Profiles of Occupational Engagement for People With Schizophrenia (POES): 1	Occupational Self-Assessment (OSA): 2	Occupational Value with predefined items (OVal-pd) (author determined value): 1		Bristol Activities of Daily Living Scale (BADLS): 1
Profiles of Occupational Engagement in People with Severe Mental Illness Productive Occupations (POES-P-SR): 1	Satisfaction with Daily Occupations (SDO): 1			Scale for Interactive Observation in Occupational Therapy (EOITO): 1
Satisfaction with Daily Occupations and Occupational Balance (SDO-OB): 1	Satisfaction with Daily Occupations and Occupational Balance (SDO-OB): 1			Functional Assessment Short Test (FAST): 1
				Functional Independence Measure (FIM): 1
				Interview for Deterioration in
				Daily Living Activities in Dementia (IDDD): 1
				Katz Index of Independence in Activities of Daily Living (KIIADL): 1
				Lawton and Brody Instrumental Activities of Daily Living (LBIADL): 1
				Performance Assessment of Self-Care Skills (PASS-CLINIC): 1
				Rehabilitation Evaluation Hall and Baker (REHAB): 1
				Social Functioning Scale (SFS): 1
				Time in work: 2
				Time use diary^a^: 1

^a^ Authors state occupational balance but no measure of subjective experience.

Four measured occupational balance (time use, range of occupations and satisfaction); five measured occupational competence (activity performance and satisfaction); three measured occupational value (value of certain occupations, either from the participant or researchers’ perspective); one measured the environment and 14 measured activity performance. Studies involving people with dementia used only measures of activity performance, whereas the mental health studies utilised the full range of measures.

Table 2 shows if citations were provided regarding the validity, reliability and sensitivity to change of the measures used. The least often cited psychometric property was sensitivity.

### Economic evaluation

Three studies reported economic evaluations. One carried out a Net Benefit Analysis and evaluated costs of mental health care and the value of work time (earnings minus costs) (Schene et al., 2007). Two studies carried out Incremental Cost Effectiveness Ratios (ICERS), and one evaluated costs associated with carers’ hours ‘doing things’ and hours ‘on duty’ in addition to intervention and control group costs (Gitlin et al., 2010). Graff and colleagues (2008) evaluated costs from a societal perspective, that is, both direct costs inside and outside the healthcare service and estimated costs for gains and losses in productivity of the caregivers.

### Process evaluation

Three papers reported aspects of process evaluation, each linked to a study reporting effectiveness separately (Gitlin et al., 2009; Voigt-Rafloff et al., 2011a; Walton et al., 2019). Only one used the term process evaluation in the title (Voigt-Rafloff et al., 2011a).

Gitlin and colleagues (2009) assessed the fidelity of delivery and contextual aspects when reporting the feasibility, acceptability and replication potential of TAP. Quantitative and qualitative data were collected from the occupational therapist interventionalists and caregiver participants to assess the: time taken to complete each intervention component; types of activities prescribed and degree of implementation and the intervention’s acceptability. However, they acknowledge the potential bias of relying on the interventionists’ perceptions.

Voigt-Radloff and colleagues (2011a) reported a process evaluation conducted alongside a multisite RCT (Voight-Radloff et al., 2011b). This included: assessing intervention delivery fidelity using data reported by the occupational therapist interventionalists; comparing the Dutch and German participant characteristics and health service use and identifying differences between the two study designs, such as using different outcome measures.

Walton and colleagues (2019) assessed the fidelity of delivery of COTiD-UK in a longitudinal observational study nested within the multisite RCT (Wenborn et al., 2021). They developed a reliable fidelity measure and used this to assess the fidelity of delivery across the sessions, sites and occupational therapists. All sessions were audio recorded if appropriate of which ten percent were purposively sampled, transcribed and coded, and the percentage of components delivered calculated. This methodology reduced the potential bias of self-reporting but has increased resource implications.

## Discussion and implications

We systematically reviewed pilot trials, RCTs and related economic evaluation or process evaluation studies of occupational therapy interventions for adult with mental health conditions or dementia. Our focus was the study methods and outcomes measured rather than intervention effectiveness. A limitation of this study is that only studies published in the English language were included; therefore, other relevant studies not published in English may have been omitted.

The results demonstrate several limitations in the quality of the current evidence. These in part reflect some common challenges in conducting RCTs of complex interventions such as occupational therapy, interventions, but also the impact of poor reporting. Researchers should attend to these common issues in their own study design to minimise bias and thus contribute stronger evidence in which readers can have confidence. We discuss the key points and make recommendations for improving methodological and reporting rigour.

### Clarity of reporting

We noted titles and abstracts did not always state the study design, population and intervention. Six did not include the words ‘occupational therapy’ or ‘occupational therapist’. Title and abstract clarity are essential to ensure studies are identified by the relevant audience. Unclear titles and abstracts risk omission from systematic reviews or meta-analysis, thus preventing the production of further, more robust evidence.

The interventions were described with varying detail about their content and development. Guidelines indicate the key elements of the intervention development process that should be transparently reported, usually in a separate paper, to make clear the evidence and underpinning theory (Duncan et al., 2020). Intervention components, delivery methods and dosage should be clearly defined prior to assessing effectiveness in an RCT (Richards, 2015). We noted a lack of description of the comparator interventions and were often not able to differentiate whether ‘usual treatment’ included occupational therapy or not.

## Methods

The majority of studies were rated as poor (*n* = 6) or fair (*n* = 13). Sometimes this was due to not reporting using standard criteria. Researchers should refer to international standards during trial design and reporting. Finding that the majority of RCTs did not report a power calculation may reflect the lack of experienced trialists or statisticians involvement, as concluded by Tokolahi et al. (2015). Research support, and research design services where accessible, should be consulted early, and early planning with a clinical trials unit is crucial to avoid introducing invalidating biases due to team inexperience.

Process evaluations were few. Trials may have negative results if intervention delivery is flawed, but in complex intervention studies, there is a heightened risk of implementation failure. This is especially so in multisite studies where the potential for variation in intervention delivery is higher (Moore et al., 2014). One aspect of process evaluation is measuring intervention fidelity. Supervision contributes to enhancing fidelity, which was reported in some studies. Only Walton et al. (2019) address intervention fidelity rigorously. To ensure that future studies are robust, embedding process evaluation activities within trials is essential.

In some studies, participants lacked diversity. There were marked imbalances in gender and few studies reported ethnicity. The gender imbalance may reflect the demographics of the sample population, but in some cases, it seems more likely to be related to study recruitment or relatability of the intervention (or both). That is, the recruitment favoured men/women or the intervention suited men/women, rather than there being an imbalanced gender profile in the sampled population. Practitioners must decide whether an intervention is relevant to people with different characteristics when applying findings to their settings. Researchers must consider how their recruitment processes operate to obtain a representative sample and report the demographics of those that decline to participate, non-completers, alongside the sample population. Readers can then draw informed conclusions about representativeness and thus, generalisability of the results.

Many problems can be avoided by applying evidence-based reporting standards for RCTs (Grant et al., 2018). Reporting in full avoids an unjustified critique of poor methodological quality. The 2001 CONSORT statement may not have been available when earlier studies were published. However, in more recent publications, its application may have assisted in clarity and quality. Many journals now require a CONSORT Checklist to be submitted as supporting information. Researchers should consider these items at the trial design stage to prevent omissions that could invalidate findings.

Minimising the risk of performance bias (masking participants as to which intervention they received) and detection bias (masking those collecting and analysing outcome data to group allocation) are important aspects of RCT design. This principle is easier to adhere to in drug trials where the active and placebo medication can be produced to look identical within the bottle. In trials of occupational therapy and other non-pharmacological interventions which require the active engagement and participation of the service user, it is difficult to mask participants. Ensuring that those collecting data remain masked to allocation is challenged by the potential for participants to disclose their experiences, allowing the assessor to infer their allocation. Whilst researchers can remind participants not to disclose this when booking appointments and before starting the data collection session, service users may have memory or cognitive impairment that affect their ability to retain this information. There may be evidence of intervention having been delivered within the home environment, for example, environmental cues installed to enhance the orientation of a person with dementia. This immediately reduces the quality of the trial when using standard assessment tools such as we did (Downs and Black, 1998). A newer adaptation of the CONSORT guidelines for psychological and social interventions, which acknowledges the challenge of masking participants and researchers, should be of value in assisting researchers planning, conducting and reporting trials of occupational therapy interventions (Grant et al., 2018).

Only three RCTs included an economic evaluation, and of those, only two carried out an Incremental Cost Effectiveness Ratio (ICER) which compares differences in costs and effectiveness of two interventions. This enables decisions to be made regarding use of an intervention in practice. Familiarity and expectation of economic evaluation may vary internationally. However, they are becoming increasingly important alongside testing effectiveness to enable funders to decide which interventions to commission. As such, having economic evaluation and a health economist funded into a research grant for RCTs of occupational therapy interventions is vital.

### Outcomes and outcome measures

Half (*n* = 13) of the studies either listed multiple primary outcomes, or did not differentiate which was the primary outcome within those listed. Most occupation-focused outcome measures in the mental health studies had limited or questionable psychometric properties in general, and in respect of the trial populations. Better quality papers make a clear statement regarding the intended primary outcome measure to be used and demonstrate that it is consistent with the aims of the intervention. Modelling intervention processes and expected outcomes are a key stage of developing a complex intervention ahead of feasibility-testing and piloting (Richards, 2015). After identifying the expected outcomes of the intervention, the primary outcome can be defined and operationalised, and a measure selected that has evidence of validity, reliability and sensitive to change with the study population. This ensures the primary outcome measure can be used to calculate the sample size needed to detect change in scores in a full RCT.

Several primary outcome measures were not clearly related to the intervention being tested for example, using a measure of psychiatric symptoms. However, there is a need to include secondary measures such as quality of life which are needed for economic evaluations, or if the funder has a particular health focus.

We noted the range of outcome measures, often with more than one used to measure the same domain. Multiple outcome measures across studies impacts on the feasibility of future meta-analyses. To reduce this variation, researchers are encouraged to select from the core outcome sets, developed for some health conditions, such as the Core Outcome Measures in Effectiveness Trials (COMET Initiative). Occupational therapists should consider these sets in the first instance to identify potentially relevant measures. Where the condition or desired outcome is not included, occupational therapists should make well justified decisions for their selection. Further, occupational therapists should contribute to developing core outcome sets.

We identified studies with methodological strengths, these were rated as good (Cook et al., 2009; Gitlin et al., 2008, 2018; Graff et al., 2006; Hees et al., 2013) or excellent (Voight-Radloff et al., 2011b; Wenborn et al., 2021). Strengths included describing the intervention aim, intended outcomes, and content/delivery schedule; assessing intervention fidelity; sample size calculated for RCTs based on the primary outcome measure; reporting psychometric properties of outcome measures used, and use of outcome measures with good psychometric properties; and more than one paper reporting the study to ensure detailed reporting.

Studies included in this review are heterogeneous in their interventions, outcomes and outcome measures, precluding comparison through meta-analysis. Meta-analysis of RCT evidence requires considerable advancements in the number, quality and reporting of RCTs. A vital first step will be addressing the limitations we have identified in RCTs of occupational therapy interventions for people with mental health conditions or dementia.

## Conclusion

We identified considerable variation in the quality of RCTs and their reporting. This limits the conclusions that can be drawn about the effectiveness of occupational therapy interventions for people with mental health conditions or dementia and precludes meta-analysis.

To address these limitations, we encourage researchers to use established guidance on developing and evaluating complex interventions, and international reporting standards to support clear reporting. Specific areas for attention are: robust development of the intervention and identification of expected outcomes, conducting feasibility and pilot studies, prior to undertaking a fully powered RCT, selecting a psychometrically robust primary outcome measure consistent with the aim of the intervention being tested and seeking to recruit as diverse a sample as feasible within the population for whom the intervention is designed. Early collaboration with clinical trials specialists and statisticians is vital to prepare the study protocol to avoid methodological errors and embed process and economic evaluations in the RCT design.

## Key findings

• There are few good quality RCTs of occupational therapy interventions for adults with mental health conditions or dementia.
• RCT methods can be improved to address limitations in the evidence.

## What the study has added

We identified methodological weaknesses in randomised controlled trials of occupational therapy interventions in mental health or dementia that researchers should avoid or minimise to strengthen the confidence in their results.

## Appendix 1 Medline search terms used

**Table:**

Search number	Search terms
S1	‘occupational therapy’ (mapped and unmapped against Medical Subject Headings (MESH) terms) OR (occupational therap* OR ‘occupational therapy intervention’ OR ‘occupational therapy treatment’ OR (occupation* OR activit* of “daily living” OR ADL OR leisure activit* OR selfcare OR “personal care” OR “everyday activit*” OR “everyday function*”) (ti/ab)
S2	(Psychiatr* OR mental health problems OR mental health difficulties OR mental illness OR mental disorder OR anxi* OR depress* OR psychosis OR psychoses OR psychotic OR schizo* OR (Eating Disorder* OR Anorexia Nervosa OR Bulimia Nervosa OR Binge Eating Disorder) OR (personality disorder* OR borderline personality OR emotionally unstable personality OR histrionic personality OR narcissistic personality OR antisocial personality OR paranoid personality OR schizoid personality OR schizotypal personality OR avoidant personality OR dependent personality OR obsessive compulsive personality) OR (Dementia OR dement* OR alzheimer* OR cognit impair* OR memory) (Ti/Ab)
S3	randomized controlled trial OR controlled clinical trial OR randomised or trial OR random* (Ti/Ab)
S4	(implement* OR process OR determinant* OR barrier* OR hinder* or obstacle* OR impediment* OR Fidelity OR process evaluation OR cost effectiveness or economic)
S5	S3 AND S4
S6	S1 AND S2 AND S3
S7	S6 OR S5
